# Carrageenan-Induced Colonic Inflammation Is Reduced in Bcl10 Null Mice and Increased in IL-10-Deficient Mice

**DOI:** 10.1155/2013/397642

**Published:** 2013-05-26

**Authors:** Sumit Bhattacharyya, Liquan Xue, Suzanne Devkota, Eugene Chang, Stephan Morris, Joanne K. Tobacman

**Affiliations:** ^1^Department of Medicine, University of Illinois at Chicago, 840 S Wood Street, CSN 440 M/C 718, Chicago, IL 60612, USA; ^2^Jesse Brown VA Medical Center, Chicago, IL 60612, USA; ^3^Department of Pathology, St. Jude Children's Research Hospital, Memphis, TN 38105, USA; ^4^Department of Medicine, University of Chicago, Chicago, IL 02215, USA; ^5^Joslin Diabetes Center, Boston, MA 60637, USA

## Abstract

The common food additive carrageenan is a known activator of inflammation in mammalian tissues and stimulates both the canonical and noncanonical pathways of NF-**κ**B activation. Exposure to low concentrations of carrageenan (10 **μ**g/mL in the water supply) has produced glucose intolerance, insulin resistance, and impaired insulin signaling in C57BL/6 mice. B-cell leukemia/lymphoma 10 (Bcl10) is a mediator of inflammatory signals from Toll-like receptor (TLR) 4 in myeloid and epithelial cells. Since the TLR4 signaling pathway is activated in diabetes and by carrageenan, we addressed systemic and intestinal inflammatory responses following carrageenan exposure in Bcl10 wild type, heterozygous, and null mice. Fecal calprotectin and circulating keratinocyte chemokine (KC), nuclear RelA and RelB, phospho(Thr559)-NF-**κ**B-inducing kinase (NIK), and phospho(Ser36)-I**κ**B**α** in the colonic epithelial cells were significantly less (*P* < 0.001) in the carrageenan-treated Bcl10 null mice than in controls. IL-10-deficient mice exposed to carrageenan in a germ-free environment showed an increase in activation of the canonical pathway of NF-**κ**B (RelA) activation, but without increase in RelB or phospho-Bcl10, and exogenous IL-10 inhibited only the canonical pathway of NF-**κ**B activation in cultured colonic cells. These findings demonstrate a Bcl10 requirement for maximum development of carrageenan-induced inflammation and lack of complete suppression by IL-10 of carrageenan-induced inflammation.

## 1. Introduction

 Carrageenans are sulfated polygalactans and are obtained from several species of red seaweed (*Rhodophyceae*). Chemically, they are composed of repeating disaccharide units and consist of sulfated or unsulfated D-galactose residues that are linked in alternating *β*-1,4 and *α*-1,3 bonds. They resemble the endogenous, human, galactose-containing sulfated glycosaminoglycans, chondroitin sulfate, and keratan sulfate, but differ by the presence of the unusual *α*-1,3 linkages [1, 2]. Carrageenans predictably induce an inflammatory response and can activate immune responses that are mediated by B-cell leukemia/lymphoma (Bcl) 10 and Toll-like receptor (TLR) 4 [3, 4]. Due to their marked chemical reactivity, carrageenans are widely used in processed foods to improve the texture of food products, although their harmful effects are well recognized [5, 6]. 

 Animal models have demonstrated that ingestion of carrageenan induces colonic inflammation, with development of inflammatory infiltrates, ulcerations, and clinical evidence of colitis [5, 6]. In studies of the effects of carrageenan on human colonic epithelial cells in cell culture, carrageenan stimulated NF-*κ*B activation and increased secretion of IL-8 by TLR4-Bcl10-dependent and Bcl10-independent pathways [3, 4, 7, 8]. Specific phosphorylations of Bcl10 were required for the NF-*κ*B responses [9], and prolongation of the effects of carrageenan in colonic epithelial cells on both noncanonical and canonical inflammatory pathways occurred due to the presence of an NF-*κ*B binding site in the Bcl10 promoter [10].

 The carrageenan-induced activation of the innate immune response in the colonic epithelial cells was attributable to its distinctive chemical structure, and, in particular, its unusual *α*-D-Gal (1→3) D-Gal link [11]. Carrageenan exposure was shown to produce NF-*κ*B activation by three distinct mechanisms, including (1) TLR4-Bcl10-IKK*γ*-IKK*β*-inhibitory factor (I*κ*B)*α*-NF-*κ*B (RelA) pathway; (2) TLR4-Bcl10-NF-*κ*B-inducing kinase (NIK)-IKK*α*-NF-*κ*B (RelB) pathway; and (3) a reactive-oxygen-species-(ROS-) mediated pathway that involves Hsp27-IKK*β*-I*κ*B*α*-NF-*κ*B (RelA) that does not require Bcl10 [3, 4, 7, 8]. These pathways indicate that Bcl10 is a key signaling molecule in the carrageenan-initiated inflammation *in vitro *in colonic epithelial cells. Recently, the impact of carrageenan on glucose tolerance, insulin resistance, and impaired insulin signaling in a mouse model was reported [12], consistent with the evidence that TLR4-mediated inflammation is involved in diabetes [13–15]. Inhibition of inflammation by Bcl10 silencing or TLR4 blocking antibody reduced the carrageenan-induced impairment of insulin signaling [12].

 Many recent investigations involve carrageenan, including almost 500 published reports in 2012. Most of these publications address the effects of anti-inflammatory treatments to alleviate carrageenan-induced inflammation, including effects of lactobacilli [16], but without specific attention to the mechanistic pathways by which carrageenan causes inflammation. Other reports consider a variety of topics, including the chemical characteristics of carrageenan as a gel in food products [17], the use of carrageenan hydrogels as therapeutic delivery vehicles [18], carrageenan as a therapy, including for the common cold [19], and thrombotic effects of carrageenan [20]. 

 This is the first report to address the requirement for Bcl10 as a mediator of carrageenan-induced inflammation in the *in vivo *animal model and extends previous findings about the mechanisms by which carrageenan produces inflammation. Prior work has shown the role of Bcl10 in normal development and function of lymphocyte populations [21]. Overexpression of Bcl10 by the translocation t(1; 14)(p22; q32) was associated with development of the mucosa-associated lymphoid tissue (MALT) lymphomas and with the constitutive overexpression of NF-*κ*B [22] by both non-canonical and canonical pathways of NF-*κ*B activation [23]. Bcl10 has been reported to mediate inflammatory cascades in colonic epithelial cells, mouse embryonic fibroblasts, hepatocytes, and human embryonic kidney 293T cells, as well as in cells of myeloid origin [3, 24–26]. Other recent work has identified Bcl10 as a link between fat (palmitate) intake and hepatic NF-*κ*B activation and insulin resistance [27], and Bcl10 was reported to coordinate the NF-*κ*B-mediated response associated with endosomal trafficking and F-actin remodeling in human macrophages [28]. Recognition of the participation of the CARMA-Bcl10-Malt1 (CBM) signalosome in vital processes in cells continues to emerge, including distinct roles of CARMA1 in immune cells and CARMA3 in epithelial/endothelial cells. The CBM complex appears to be a signaling platform, enabling the integration of effects from receptors with downstream cascades, including effects from G-protein coupled receptors [29]. The CBM complex was shown to promote angiotensin II-dependent vascular inflammation and atherogenesis, and these effects were reported to be reduced in Bcl10-deficient mice [30]. The protein A20, also known as tumor necrosis factor alpha-induced protein 3 (TNFAIP3), acts as a negative regulator of the Bcl10-CARMA interactions in both the lymphoid and nonlymphoid cells due to its effects as a ubiquitin ligase and deubiquitinase [31]. 

 In this report, we evaluate the effect of carrageenan in Bcl10 null mice and the pathways of carrageenan-induced inflammation mediated by activation of canonical and non-canonical cascades. Also, since the microbiome and IL-10 are both widely recognized to impact intestinal inflammation, the effects of the germ-free environment and of exogenous IL-10 on carrageenan-induced inflammation are evaluated in order to provide additional insights into the mechanisms and mediators by which carrageenan provokes inflammation.

## 2. Materials and Methods

### 2.1. Animal Care and Carrageenan Exposure

 Eight-week-old male C57BL/6J mice were purchased (Jackson Laboratories, Bar Harbor, Maine, USA) and housed in the Veterinary Medicine Unit (VMU) at the Jesse Brown VA Medical Center (JBVAMC, Chicago, IL, USA). Principles of laboratory animal care were followed, and all procedures were approved by the Animal Care Committee. Adult mice were fed a standard diet and maintained in individual cages with routine light-dark cycles. After acclimatizing to the environment, the water supply was changed to ddH_2_O with undegraded carrageenan (*λ*-*κ* carrageenan 10 mg/L; Sigma Chemical Co., St Louis, MO, USA; *n* = 6) or without carrageenan (*n* = 6) for ~12 weeks. Weight and water consumption were measured regularly. Bcl10 null (*n* = 8), heterozygote (*n* = 3), and wild type (*n* = 6) mice were bred and genotyped at St. Jude Children's Research Hospital by study investigators (Stephen Morris and Liquan Xue) [21–23]. Adult mice were shipped to the JBVAMC VMU. Following quarantine, adult mice were treated with carrageenan in their water supply. Stool was collected, and mice were euthanized after receiving carrageenan for ~14 weeks. Total carrageenan intake averaged ~11.5 mg/30 g mouse. Blood was collected by cardiac puncture at the time of euthanasia, and organs were immediately harvested and frozen. Carrageenan (10 mg/L × 19 days + 100 mg/L × 9 days) was also given in the water supply to adult C57BL/6 mice (*n* = 5) and IL-10-deficient mice (*n* = 4) in the germ-free facility at the University of Chicago under an approved protocol and direction of study investigators (Eugene Chang and Suzanne Devkota) [32]. Control littermates were not given carrageenan. Mice were euthanized by carbon dioxide and exsanguination by cardiac puncture, blood was collected, and stool and organs were harvested and frozen pending further studies. Germ-free status was assessed by detection of 16S rRNA by PCR from stool and cecal contents.

### 2.2. NCM460 Cells in Tissue Culture

NCM460 cells (INCELL, San Antonio, TX, USA) were grown in M3:10A media under the established conditions as previously described [3, 4, 33]. Bcl10 siRNA was used as previously described [3, 4]. Cells were exposed to *λ*-CGN (1 mg/L × 24 h) in the presence or absence of bioactive, exogenous IL-10 (Sigma-Aldrich Chemical Co., St. Louis, MO, USA 10 or 20 *μ*g/L × 24 h). At the end of the treatment, spent media were collected from control and treated wells and stored at −80°C. Total cell protein was measured by the BCA protein assay kit (Pierce, Rockford, IL, USA) using BSA as the standard.

### 2.3. Measurement of Bcl10 and Phospho-Bcl10

 Bcl10 protein in the colonic epithelium of control and carrageenan-fed mice was determined by a solid-phase ELISA reported previously [34]. To determine phospho-Bcl10 in mouse colonic epithelium and in control and CGN-treated NCM460 cells, Bcl10 in the samples was captured in a 96-well ELISA plate that was precoated with Bcl10 antibody, sandwiched by a phospho(Ser138)-Bcl10 second antibody, and then detected by a specific anti-rabbit IgG and hydrogen peroxide-TMB chromogenic substrate [8, 9]. Intensity of the developed color was proportional to the quantity of phospho-Bcl10 in the sample, and optical density values were determined (FLUOstar, BMG, Cary, NC, USA) and normalized using the total cell protein determined by a protein assay kit (Pierce). Results were expressed as percent of total Bcl10 in the untreated control.

### 2.4. ELISA for Phospho-I*κ*B*α* in Colon of Carrageenan-Exposed Mice and in NCM460 Cells

 Commercial sandwich ELISA for phospho(Ser32)-I*κ*B*α* (Cell Signaling Technology, Inc., Danvers, MA, USA) was used to determine the phosphorylation of I*κ*B*α* that was produced following carrageenan exposure in the mouse colonic epithelial tissue and in NCM460 cells [3, 4]. Briefly, the I*κ*B*α* in the cell extracts was captured in a 96-well ELISA plate that was precoated with mouse monoclonal antibody against I*κ*B*α*. Phospho-I*κ*B*α* was determined by a specific phospho(Ser32)-I*κ*B*α* antibody and detected by horseradish peroxidase- (HRP-) conjugated secondary antibody and hydrogen peroxide-tetramethylbenzidine (TMB) chromogenic substrate. Phospho-I*κ*B*α* was expressed as percent of total I*κ*B*α* in the untreated control.

### 2.5. Oligonucleotide-Based ELISA for Measurement of Nuclear RelA and RelB

 Nuclear extracts were prepared from either the colonic epithelial cells of control and carrageenan-fed mice or carrageenan-treated and control NCM460 cells by a nuclear extraction kit (Active Motif, Carlsbad, CA, USA). Activated NF-*κ*Bs, including RelA and RelB, were determined by oligonucleotide-based ELISA (Active Motif), as previously described [8]. Treated and control samples were incubated in 96-well microtiter plates that were coated with the NF-*κ*B consensus nucleotide sequence (5′-GGGACTTTCC-3′). NF-*κ*B from the samples attached to the wells and was captured by specific antibody to either RelA or RelB. The extent of binding of the antibody was detected by anti-rabbit-HRP-conjugated IgG, and color was developed with hydrogen peroxide-TMB chromogenic substrate. Intensity of the developed color was proportional to the quantity of either RelA or RelB in the sample. Specificity of the NF-*κ*B binding to the nucleotide sequence was determined by the premixing of either free consensus nucleotide or mutated consensus nucleotide to the nuclear extract sample before adding the sample to the well. The optical density values were normalized using the total cell protein determined by a protein assay kit (Pierce), and results were expressed as percent of the unexposed control.

### 2.6. ELISA for Measurement of KC and Other Cytokines in Mouse Serum

 Serum keratinocyte chemokine (KC), the mouse homolog of IL-8, was detected in control and carrageenan-exposed Bcl10 wild type, heterozygotic, and null mice, and untreated control C57BL/6J mice (total *n* = 12) by DuoSet ELISA (R&D Systems, Minneapolis, MN, USA), as previously described [4]. In addition to the KC ELISA, a custom cytokine ELISA array that included TNF-*α*, IL-6, IFN*γ*, IL-1*β*, IL-10, IL-12, MCP- (monocyte chemotactic protein-) 1, IL-12, and IL-23 was prepared (Signosis, Sunnyvale, CA, USA) and serum of carrageenan-treated Bcl10 wild type, heterozygotic, null mice, and untreated control C57BL/6J mice (total *n* = 12) was tested. Hydrogen peroxide-TMB chromogenic substrate was used to develop the color; color development was stopped with a stop solution, and the concentrations of the cytokines were directly proportional to the intensity of color measured spectrophotometrically at 450 nm in an ELISA plate reader (FLUOstar; BMG). KC concentrations were extrapolated from a standard curve. Other chemokines and cytokines were expressed as percent of control, based on determinations from mice unexposed to carrageenan. Technical duplicates were performed for all measurements, and the mean of the two readings was used in subsequent comparisons.

### 2.7. Measurement of Fecal Calprotectin

 Fecal calprotectin was measured in stools of control and carrageenan-treated mice by ELISA (Alpco Diagnostics, Salem, NH, USA) [35]. Feces were collected from control and treated animals at the time of euthanasia, and recommended procedures were followed. Stool samples were extracted in the supplied extraction buffer, centrifuged for 5 minutes at 13,000 g, then used in the sandwich ELISA. TMB was used for the development of color, which was read at an OD of 450 nm in a plate reader (FLUOstar) and compared to the standard curve.

### 2.8. Western Blots for Phospho(Thr559)-NIK and Phospho(Thr184)-Tak1

 Tissue homogenates were prepared from colonic epithelial tissue of control and carrageenan-exposed mice in lysis buffer (Cell Signaling Technology, Inc., Danvers, MA, USA) with protease and phosphatase inhibitors (Halt Protease and Phosphatase Inhibitor Cocktail, Thermo Scientific, Pittsburgh, PA, USA). Western blots were performed on 10% SDS gels with commercial antibodies to phospho(Thr559)-NIK, phospho(Thr184)-TAK, and *β*-actin (Santa Cruz Biotechnology, Santa Cruz, CA, USA). Immunoreactive bands were visualized using enhanced chemiluminescence (Amersham, GE Healthcare, Piscataway, NJ, USA). ImageJ software (NIH, Bethesda, MD, USA) was used for densitometry. The density of the protein of interest was normalized with the density of *β*-actin or tubulin from the same specimen, and density of treated and control samples was compared.

### 2.9. Histopathology of Colon from Bcl10 Wild Type and Null Mice Following Carrageenan

 Intestine and other organs from control and carrageenan-fed mice were sampled, processed in 10% neutral-buffered formalin and paraffin blocks were made by standard procedures. The paraffin blocks were sectioned, and tissues were stained with hematoxylin and eosin by the Veterinary Diagnostic Laboratory of the University of Illinois (Urbana, IL, USA). Slides of duodenum, jejunum, ileum, cecum, colon, and rectum from three Bcl10 wild type, three Bcl10 heterozygotic, and three Bcl10 null mice exposed to carrageenan were analyzed by a veterinary pathologist (Susan Ball-Kell Global Path Imaging, Germantown Hills, IL, USA), who examined blindly the tissue sections from the gastrointestinal sites and recorded on a standardized scale ratings of inflammation, corresponding to the Ameho criteria with six gradations [36]. These grades were: occasional = 0–3 inflammatory cells/hpf, grade 0 of normal; normal to trace inflammatory infiltrate = 4–10 inflammatory cells/hpf, grade “1” or small; mild inflammatory infiltrate = 11–20 inflammatory cells/hpf, grade “2” or small-medium; mild-moderate inflammatory infiltrate = 21–30 inflammatory cells/hpf, grade “3” or medium; moderate inflammatory infiltrate = 31–40 inflammatory cells/hpf, grade “4” or medium-large; moderate to severe inflammatory infiltrate = 41–50 cells/hpf, grade “5” or large; severe inflammatory infiltrate =>50 inflammatory cells/hpf, grade “6” or marked. Photomicrographs were taken with 20x objective.

### 2.10. Statistical Analysis

 Data were analyzed using InStat3 software (GraphPad, La Jolla, CA, USA). Mean values ± standard deviation (S.D.) were calculated, and differences between carrageenan-treated and control results were compared by one-way ANOVA with Tukey-Kramer post-test for multiple comparisons or by unpaired *t*-tests, two-tailed. Unless stated otherwise, the one-way ANOVA test was used for the reported comparisons. All measurements are the result of independent experiments with at least three biological samples and two technical replicates, with the exception of the control IL-10-deficient mice in which the *n* was 2. In the figures, * is for *P* ≤ 0.05, ** is for *P* ≤ 0.01, and *** is for *P* ≤ 0.001.

## 3. Results

### 3.1. Histopathology of Colonic Tissue Following Carrageenan Exposure

**Figure 1:**
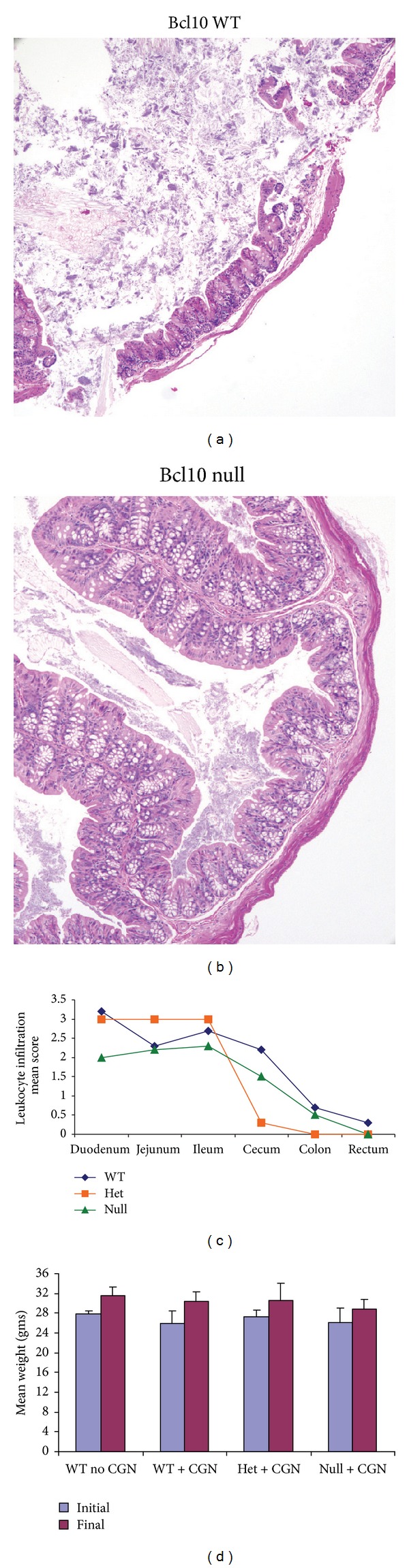
Histopathology of intestinal tissue in Bcl10 wild type and null mice following carrageenan exposure. (a), (b) Bcl10 WT, heterozygous, and null mice were sacrificed after ingestion of carrageenan (10 *μ*g/mL) added in their water for 30 days. Histopathology of H&E sections of cecum demonstrated disruption of the mucosa in a WT mouse, with no similar findings in the Bcl10 null mice, consistent with reduced inflammation in the absence of Bcl10 [WT: wild type]. (c) Throughout the mouse intestine, the extent of inflammatory infiltrate, including granulocytes, lymphocytes, and plasma cells, was greater in the Bcl10 WT mice, than in the Bcl10 null mice. The histopathology was scored for 3 WT, 3 heterozygous, and 3 null mice, and the mean scores for leukocyte infiltration for each site are compared. The scores for the WT mice are higher at each site than for the null mice, although the differences are not statistically significant. The extent of inflammatory infiltrate was significantly greater in the small intestine than in the colon and rectum for each of the groups (*P* < 0.01). Cecal inflammation, including leukocyte infiltration and edema, was greater in the WT mice than in the heterozygous or null mice. (d) The mouse weights at the onset of the carrageenan exposure and at termination are presented and indicate no significant differences in weight and slight weight gain in all groups. (*n* for no CGN control=3; *n* for WT with CGN = 6; *n* for het with CGN = 3; *n* for null with CGN = 8) (WT: wild type; het: heterozygous; CGN: carrageenan).

 Following exposure to ~18 mg of carrageenan in the water supply over 14 weeks, Bcl10 wild type, heterozygotic, and null mice and unexposed C57BL/6J mice were euthanized, and the intestine and other organs were excised. No gross differences in the length of the intestine or in macroscopic lesions were evident. The intestine was immersed in 10% neutral-buffered formalin, embedded in paraffin, sectioned, and stained with hematoxylin and eosin. Disruption of the mucosal surface with hemorrhage and inflammatory infiltrate was seen in the cecal tissue of one Bcl10 wild type mouse (Figure 1(a)), but not in any of the other mice (Figure 1(b)). No macroscopic abnormalities were observed, and the intestine did not appear shortened. 

 The intestines were evaluated for microscopic changes using a six-point scale based on the extent of leukocyte infiltration, as described in Section 2. Throughout the small and large intestines, the wild-type mice had more leukocyte infiltration, including granulocytes, plasma cells, and lymphocytes, than the Bcl10 null mice, consistent with increased inflammation when Bcl10 was present (Figure 1(c)). The differences in the scores among the Bcl10 WT, heterozygous, and null mice were not statistically significant. Leukocyte infiltration was greater in the small intestine than in the colon and rectum for all of the mice (*P* < 0.01). The Bcl10 WT mice had more cecal infiltration than the null or heterozygous mice. No edema was seen in the null mice, in contrast to edema of the lamina propria and submucosa in the cecum and rectum of the heterozygous and WT mice. The initial and final weights of the mice indicated no significant differences among the groups (Figure 1(d)). 

### 3.2. KC and Fecal Calprotectin Increased Less in Bcl10 Null Mice Following Carrageenan

**Figure 2:**
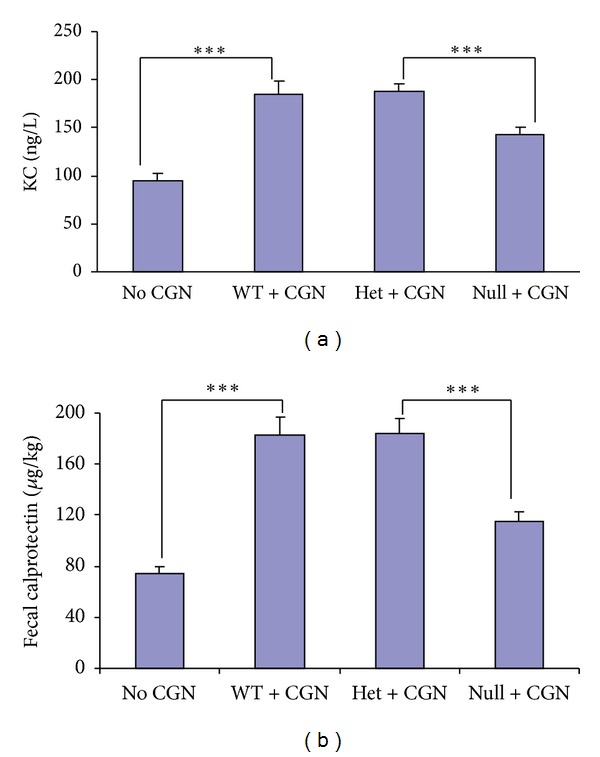
Carrageenan-induced increases in KC and calprotectin are less in Bcl10 null mice. (a) KC, the mouse homolog of IL-8, increased less in the Bcl10 null mice (*n* = 8) than in the wild type (*n* = 6) or heterozygous (*n* = 3) mice following carrageenan to ~77% of the higher value (142.2 ± 9.0 ng/L versus 188.5 ± 6.4 ng/L; *P* < 0.001). (b) Fecal calprotectin, an indicator of colonic inflammation, also increased less following carrageenan in the Bcl10 null mice than in the wild type or heterozygous mice, to 115.1 ± 8.0 *μ*g/kg versus 182.7 ± 14.3 *μ*g/kg in the wild type mice (*P* < 0.001). CGN: carrageenan; KC: keratinocyte chemokine; WT: wild type; het: heterozygous.

 The serum keratinocyte chemokine (KC), the mouse homolog of IL-8, increased to ~2.0 times the no carrageenan baseline value in the wild type and heterozygous Bcl10 mice (*P* < 0.001). The increase in serum KC following carrageenan was less (to ~1.5 times the baseline) in the Bcl10 null mice than in the Bcl10 wild type or heterozygous mice (*P* < 0.001) (Figure 2(a)). Similarly, the increase in fecal calprotectin following carrageenan was less in the Bcl10 null mice than in the Bcl10 wild type or heterozygous mice (*P* < 0.001) (Figure 2(b)). These findings are consistent with reduced intestinal and systemic inflammation in the absence of Bcl10. 

### 3.3. Increased Serum IL-6 and MCP-1 Following Carrageenan Exposure in Bcl10 Mice

**Figure 3:**
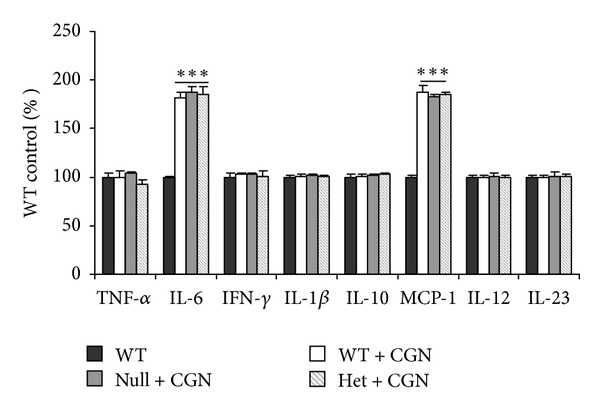
Serum IL-6 and MCP-1 increase post-carrageenan exposure, and increases are similar in Bcl10 WT, heterozygous, and null mice. Carrageenan exposure produced significant increases in serum levels of IL-6 and MCP-1 in the WT, heterozygous, and null mice (*P* < 0.001), compared to the unexposed controls. WT, heterozygotes, and null mice serum levels of MCP-1 and IL-6 both increased ~85%. Serum levels of TNF-*α*, IFN*γ*, IL-1*β*, IL-10, IL-12, and IL-23 did not change.

Serum was collected from the Bcl10 WT, heterozygous, and null mice after exposure to carrageenan for 14 weeks. Cytokine ELISA was performed and indicated significant and similar increases following carrageenan in MCP-1 and IL-6 in the Bcl10 WT, heterozygous, and control mice, compared to controls that were not exposed to carrageenan (*P* < 0.001) (Figure 3). Serum TNF*α*, IFN*γ*, IL-1*β*, IL-10, IL-12, and IL-23 were unchanged following carrageenan. 

### 3.4. Activation of NF-*κ*B RelA Reduced and of RelB Inhibited in Bcl10 Null Mice Following Carrageenan

**Figure 4:**
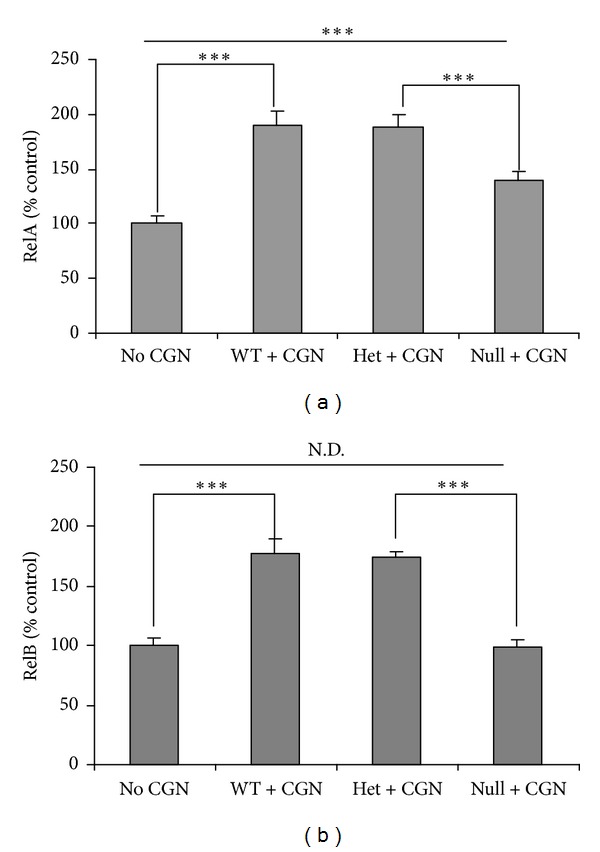
Carrageenan-induced increase in RelA is reduced and is absent in RelB in Bcl10 null mice. (a) Nuclear RelA, measured by oligonucleotide assay, increased by ~89% in the wild type and heterozygous mice following carrageenan but only by about 40% in the Bcl10 null mice (*P* < 0.001). (b) In contrast, the increase in RelB was completely inhibited in the Bcl10 null mice but increased by ~75% in the heterozygous and wild type mice following carrageenan (*P* < 0.001). CGN: carrageenan; WT: wild-type; Het: heterozygous; N.D.: no difference.

 Measurements of activated nuclear RelA and RelB in the colonic epithelium of carrageenan-exposed mice and controls revealed that activation of RelA was significantly increased in wild type, heterozygous, and Bcl10 null mice after carrageenan exposure, but the increase was significantly less in the Bcl10 null mice (*P* < 0.001) (Figure 4(a)). Activated RelB was increased ~75% in wild type and heterozygous mice, but this increase was completely inhibited in the Bcl10 null mice (*P* < 0.001) (Figure 4(b)).

### 3.5. Phosphorylations of Bcl10 and of I*κ*B*α*

**Figure 5:**
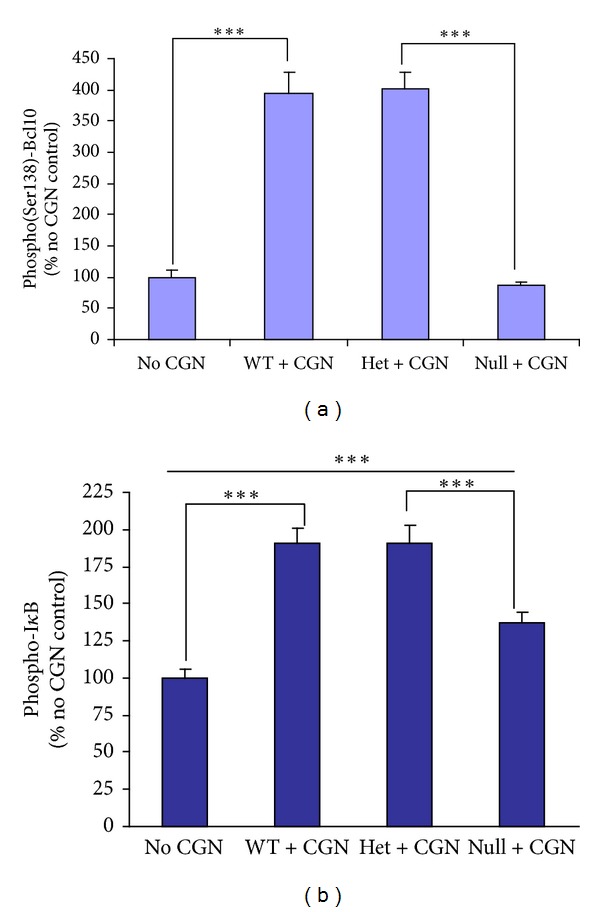
Phosphorylation of Bcl10 is absent and of I*κ*B*α* is reduced in Bcl10 null mice following carrageenan. (a) Phospho(Ser138)-Bcl10 was increased to ~400% of the no carrageenan baseline when the Bcl10 wild type and heterozygous mice were exposed to carrageenan (*P* < 0.001) but not in the Bcl10 null mice. (b) The increase in phospho(Ser32)-I*κ*B*α* is less in the Bcl10 null mice, compared to the wild type and heterozygous mice (increase of ~37% versus ~91%), but the increase from the baseline no carrageenan value is also significant (*P* < 0.001). CGN: carrageenan; WT: wild type; Het: heterozygous.

 Phosphorylations of Bcl10 and I*κ*B*α* were measured by ELISAs in colonic epithelial tissue, following exposure to carrageenan in the Bcl10 transgenic mice and in control, untreated C57BL/6 mice. Four fold increases in phospho(Ser138)-Bcl10 were measured in the carrageenan-exposed Bcl10 wild type and heterozygous mice (*P* < 0.001), but not in the Bcl10 null mice, as anticipated (Figure 5(a)). Phospho(Ser32)-I*κ*B*α* level was measured by ELISA in the colonic epithelial tissue of the carrageenan-treated Bcl10 wild type, heterozygous, and homozygous null mice and control, untreated C57BL/6 mice. Following carrageenan, phospho-I*κ*B*α* increased to almost twice the level of the untreated control value in the wild type and heterozygous mice, but in the Bcl10 null mice the increase was nearly 50% less (*P* < 0.001) (Figure 5(b)).

### 3.6. Phosphorylations of NIK and of Tak1

**Figure 6:**
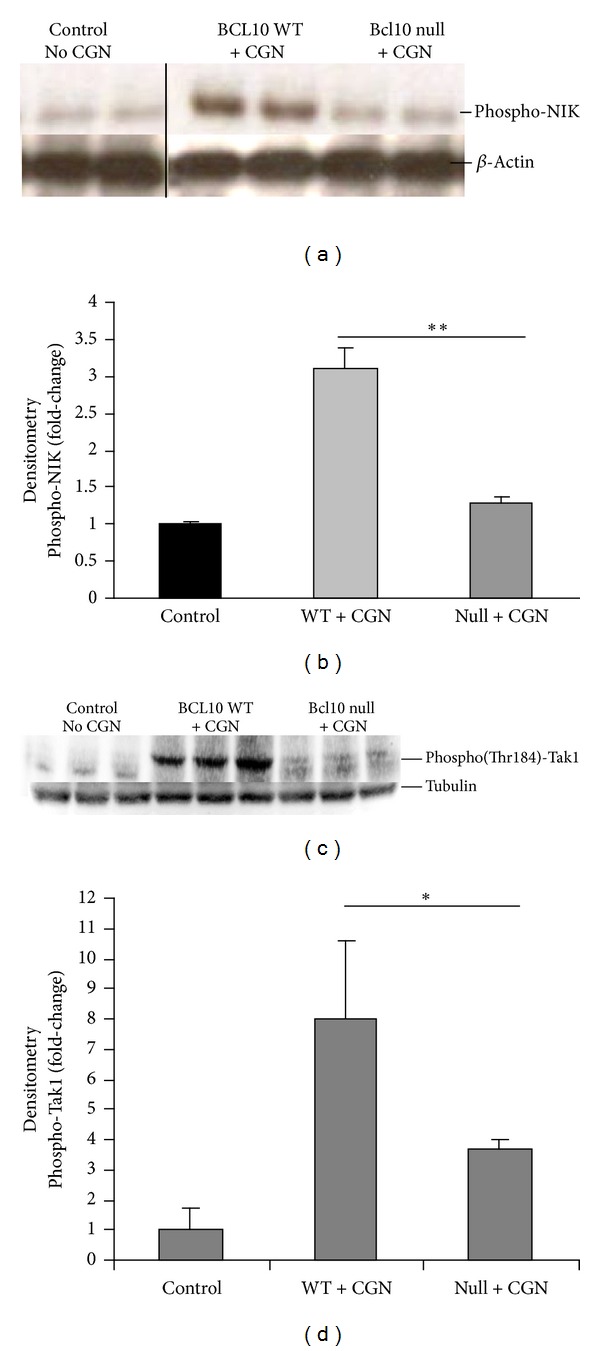
Different responses of phospho(Thr559)-NIK and of phospho(Thr184)-Tak1 following carrageenan in Bcl10 null mice. (a) The immunoblot shows that phospho-NIK does not increase following exposure to carrageenan in the Bcl10 null mice, in contrast to the finding in the wild type mice. (b) Densitometry confirms the increase in phospho-NIK in the Bcl10 WT mice treated with carrageenan compared to the Bcl10 null mice (*P* = 0.01, unpaired *t*-test, two-tailed). (c) In contrast, phospho-Tak1(Thr184) was somewhat increased in the Bcl10 null mice's intestinal tissue, although less than in the wild type mouse tissue. (d) Densitometry confirms the visual impression that the phospho-Tak1 is significantly reduced in the Bcl10 null versus Bcl10 WT mouse following CGN exposure (*P* < 0.05, unpaired *t*-test, two-tailed). CGN: carrageenan; WT: wild type; NIK: NF-*κ*B inducing kinase; Tak: TGF-*β* activated kinase.

 To compare the activation of the canonical and non-canonical pathways in the Bcl10 transgenic mice, phospho-NIK, which is required for RelB activation by the non-canonical pathway, and phospho-Tak, which is involved in the canonical pathway, were determined. Western blots of colonic epithelial tissue from untreated C57/BL6 mice and carrageenan-treated Bcl10 wild type and null mice indicated no increase in phospho(Thr559)-NIK in the Bcl10 null mice, in contrast to the wild type mice (Figure 6(a)). Densitometry confirms the visual impression of the greater increase in phospho-NIK in the Bcl10 wild type than in the Bcl10 null mice (*P* = 0.01) (Figure 6(b)). The carrageenan-induced increase in phospho(Thr184)-Tak in the mouse colonic epithelium was significantly greater in the Bcl10 wild type mice than in the Bcl10 null mice (Figure 6(c)), compared to the untreated control (*P* < 0.05 by densitometry) (Figure 6(d)). Absence of phospho(Thr559)-NIK in the Bcl10 null mice is consistent with the requirement for Bcl10 in the non-canonical RelB pathway of NF-*κ*B activation by carrageenan. In the Bcl10 null mice, the increase in phospho(Thr184)-Tak was less than in the Bcl10 wild type mice, consistent with the presence of both Bcl10-dependent and Bcl10-independent pathways by which carrageenan increases phospho(Thr184) Tak1. 

### 3.7. Effects of Carrageenan in the Germ-Free Environment and in IL-10 Null Mice

**Figure 7:**
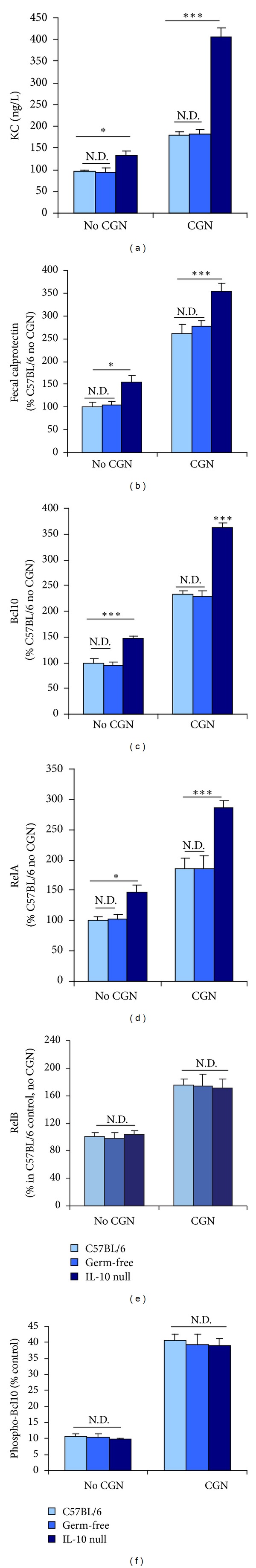
Effects of carrageenan exposure in IL-10-deficient mice compared to C57BL/6 control in germ-free environment and in standard housing. (a) KC was not different in the C57BL/6 germ-free mice versus mice with standard housing at baseline or following carrageenan exposure. In the IL-10-deficient mice, KC was greater at baseline (*P* < 0.05) and following carrageenan (*P* < 0.001) than in the control mice, and the increase in KC was greater in the IL-10-deficient mice than in the controls (~271 ng/L versus ~87 ng/L). (b) Fecal calprotectin was not different in the germ-free mice versus the standard housing control mice with or without carrageenan exposure. Fecal calprotectin was greater in the IL-10-deficient mice at baseline (*P* < 0.05) and following CGN (*P* < 0.001). (c) Bcl10 was similar in the C57BL/6 mice in the germ-free environment as in standard housing. Baseline Bcl10 was significantly greater in the IL-10 null mice than in the controls (*P* < 0.001), and increased more in the IL-10-deficient mice (~229% versus ~144%) following carrageenan (*P* < 0.001) than in the control mice. (d) The germ-free environment did not affect the RelA results in the C57BL/6 mice. Baseline RelA was significantly greater in the IL-10 null mice than in the control C57Bl/6 mice (*P* < 0.05) and increased more following carrageenan in the IL-10-deficient mice than in the controls (*P* < 0.001). (e) Carrageenan-induced increase in RelB was unaffected by IL-10 deficiency. (f) Carrageenan-induced increase in phospho(Ser138)-Bcl10 was unaffected by IL-10 deficiency or the germ-free environment. CGN: carrageenan; N.D.: no difference.

C57BL/6 mice were exposed to carrageenan in the germ-free environment. The germ-free environment did not affect the responses of KC (Figure 7(a)), fecal calprotectin (Figure 7(b)), Bcl10 (Figure 7(c)), RelA (Figure 7(d)), RelB (Figure 7(e)), or phospho-Bcl10 (Figure 7(f)). These findings enabled the comparison between the C57BL/6 mice and the IL-10 mice which require the germ-free environment due to their compromised immunity.

 In the IL-10 null mice in the germ-free environment, the baseline values without carrageenan of KC (*P* < 0.05) (Figure 7(a)), fecal calprotectin (*P* < 0.05) (Figure 7(b)), Bcl10 (*P* < 0.001) (Figure 7(c)), and RelA (*P* < 0.05) (Figure 7(d)) were all significantly greater than in the C57BL/6 mice. Carrageenan exposure produced larger increases in these measures in the IL-10 null mice than in the C57BL/6 mice (*P* < 0.001). In contrast, the baseline values and carrageenan-stimulated increases in RelB (Figure 7(e)) and phospho-Bcl10 (Figure 7(f)) were similar in the IL-10-deficient mice, in the C57BL/6 mice, and in the germ-free environment, indicating that IL-10 deficiency did not affect activation of the non-canonical NF-*κ*B pathway. 

### 3.8. Inhibition of the Carrageenan-Activated Canonical, but Not the Non-Canonical NF-*κ*B Pathway by Exogenous IL-10

**Figure 8:**
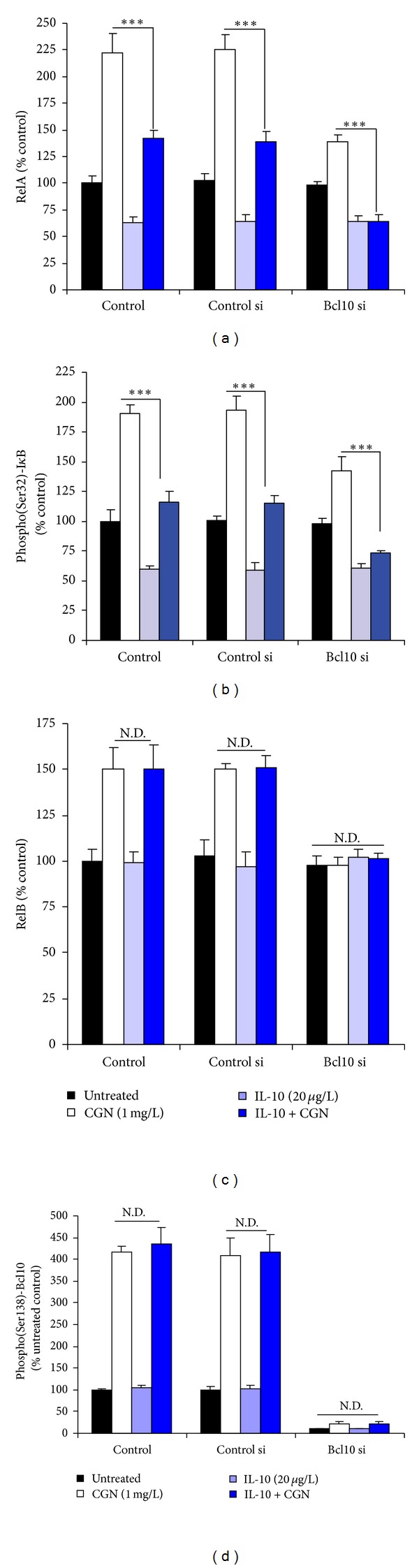
In NCM460 cells, exogenous IL-10 inhibits the canonical but not the non-canonical NF-*κ*B pathway that is activated by carrageenan and mediated by Bcl10. (a) Increase in RelA following exposure to carrageenan was partially inhibited by exogenous IL-10 in the NCM460 cells. When Bcl10 was silenced by siRNA, the increase in RelA was reduced and completely inhibited by IL-10 (20 *μ*g/L × 24 h). (b) The increase in phospho(Ser32)-I*κ*B*α* was partially inhibited by exogenous IL-10 in the control and control siRNA cells. The increase was less when Bcl10 was silenced and was almost completely inhibited by exogenous IL-10. (c) In contrast to the above findings, the increase in RelB was unaffected by exogenous IL-10. (d) Phospho(Ser138)-Bcl10 increased to over four times the baseline in the control and control siRNA cells, and the increases were not inhibited by exogenous IL-10. ND: no difference; IL-10: interleukin-10; si: small interfering siRNA; CGN: carrageenan.

 When human colonic NCM460 cells were exposed to carrageenan and exogenous IL-10, the RelA (Figure 8(a)) and phospho-I*κ*B*α* (Figure 8(b)) responses to carrageenan declined, but the RelB (Figure 8(c)) and phospho-Bcl10 (Figure 8(d)) responses were unaffected. These results are consistent with the *in vivo *findings above and indicate that IL-10 acts only on the canonical pathway of NF-*κ*B activation that involves phospho-I*κ*B*α* and RelA. Phospho-Bcl10 and RelB, which participate in the non-canonical pathway, are not inhibited, since the anti-inflammatory cytokine IL-10 inhibits the activation of the phospho-I*κ*B*α*-mediated pathway.

## 4. Discussion

 BCL10 has emerged as a key molecule in the activation of the inflammatory cascade in epithelial cells as well as in immune cells [3, 24–26]. Recent investigations in colonic epithelial cells demonstrated the requirement for Bcl10 Ser138 phosphorylation for the activation of the non-canonical pathway of NF-*κ*B activation [9]. The identification of an NF-*κ*B-Bcl10 loop by which an NF-*κ*B binding site in the Bcl10 promoter can lead to transcriptional effects on Bcl10 expression presents a mechanism by which carrageenan, and other Bcl10 stimulators, can lead to prolonged stimulation of inflammatory responses [10].

 The studies in this report demonstrate a profound impact of Bcl10-mediated inflammation on the mammalian intestine. In Bcl10 null mice, histopathology following exposure to carrageenan showed less intense inflammatory response than in the wild type mice. Decline in activation of both canonical and non-canonical NF-*κ*B activation occurred when Bcl10 was absent, as indicated by reductions in RelA and RelB. In contrast, studies in IL-10-deficient mice treated with carrageenan demonstrated effects on the canonical, but not the non-canonical pathway of NF-*κ*B activation. Complementary studies in NCM460 cells with exogenous IL-10 following exposure to carrageenan showed effects of IL-10 on RelA, but not on RelB activation. IL-10 gene-knockout mice are known to develop a colitis that resembles IBD [37], and IL-10 gene and IL-10 receptor mutations have been associated with clinical IBD [38]. However, IL-10 therapy has not become as useful clinically as anticipated [39]. By the demonstration of Bcl10-mediated non-canonical inflammation in the IL-10 deficient mouse model, the study findings suggest that specific therapeutic targeting of the non-canonical pathway of NF-*κ*B activation, as well as targeting of the canonical pathway by IL-10, may lead to improved clinical outcomes. Recent findings that oral *Lactobacillus* significantly reduced carrageenan-induced paw edema and downregulated TNF-*α* and upregulated IL-10 [16] are suggestive that combined interventions, which impact upon both canonical and non-canonical NF-*κ*B signaling pathways, may lead to improved responses.

 The study findings include data that indicate that carrageenan-induced inflammation was not suppressed in the germ-free environment. This result suggests that the fecal microflora, as constituted in the C57BL/6 mice under standard conditions, either do not modify carrageenan or do not modify carrageenan in any way that enhances inflammation. The response to *Lactobacillus* noted above suggests that the specific microflora can affect the response to carrageenan, but the effect appears to be indirect, through changes in cytokines or circulating immune cells.

 Carrageenan has been widely used in the laboratory for decades to stimulate inflammation in animal models and in cell-based experiments [5, 6]. The animal models have included injection of carrageenan directly into rat hind paw, pleura, peritoneum, bursa, subcutaneous air bleb, or large joints, and oral ingestion to induce colonic inflammation. The signaling mechanisms by which carrageenan induces inflammation have been investigated [3, 4, 7, 8], and the mediators by which carrageenan exposure can lead to systemic effects have been reported [3, 4, 7, 8, 40–42]. The tissue and systemic effects of carrageenan are also mediated by effects on immune cells, including neutrophils, macrophages, and lymphocytes. Mediation of carrageenan-induced inflammation by an inflammatory cell infiltrate was recognized in early experiments in which macrophages in the lamina propria, spleen, and liver were observed to contain fibrillar inclusions and/or have metachromatic staining following oral exposure to carrageenan [43, 44]. Extracolonic manifestations of carrageenan exposure leading to inflammation and impaired insulin signaling in the mouse liver were associated with glucose intolerance and insulin resistance in recent experiments [12]. 

 In B and T cells, and in epithelial cells, Bcl10 is involved in NF-*κ*B signal transduction involving TLR4 and TAK1 [3, 4, 24–26, 45–47]. The assembly of the lipid raft-associated CARMA-Bcl10-MALT1 (CBM) signalosome involves CARMA1 in the immune cells and CARMA3 in nonimmune cells [23, 48, 49]. The CBM complex promotes the activation and ubiquitination of IKK*γ* (NEMO), the regulatory component of the IKK signalosome, leading to RelA nuclear translocation. Additional experiments that better characterize the components of the inflammatory infiltrate in the intestine by FACS analysis and that study chimeric mice in which the effects of Bcl10 deficiency in the colonic epithelial cells versus the bone marrow can be differentiated will clarify specific subsets that mediate the response to carrageenan. Immune effects of carrageenan have been recognized for decades [43], and increased attention to the role of Bcl10 provides an opportunity to further differentiate the specific signaling mediators and mechanisms involved in innate and adaptive immunity. 

 In the experiments in this report, the Bcl10 transgenic mice, weighing about 30 grams, received carrageenan in their water supply at a concentration of 10 *μ*g/mL and drank about 5 mL/day, for a dose of 50 *μ*g/30 g/day (=~1.7 *μ*g/g/day = ~1.7 mg/kg/day). The reported average daily intake of carrageenan in the typical Western diet is reported to be 250 mg/day [50], corresponding to 250 mg/60 kg/day (=~4.2 mg/kg/day), considerably more than the amount ingested by the mice. Greater understanding of the mediators by which carrageenan exerts inflammatory effects *in vivo *and consideration of interventions that might inhibit carrageenan-induced inflammation are relevant to human diseases, such as diabetes and atherosclerosis in which inflammation is involved, and may yield new insights into the role of inflammation on initiation and progression of human diseases.
